# Bonsecamin: A New Cyclic Pentapeptide Discovered through Heterologous Expression of a Cryptic Gene Cluster

**DOI:** 10.3390/microorganisms9081640

**Published:** 2021-07-31

**Authors:** Constanze Lasch, Marc Stierhof, Marta Rodríguez Estévez, Maksym Myronovskyi, Josef Zapp, Andriy Luzhetskyy

**Affiliations:** 1 Department of Pharmaceutical Biotechnology, Saarland University, 66123 Saarbruecken, Germany; constanze.lasch@uni-saarland.de (C.L.); m.stierhof@t-online.de (M.S.); marta.rodriguezestevez@uni-saarland.de (M.R.E.); maksym.myronovskyi@uni-saarland.de (M.M.); 2 Department of Pharmaceutical Biology, Saarland University, 66123 Saarbruecken, Germany; j.zapp@mx.uni-saarland.de; 3 Helmholtz Institute for Pharmaceutical Research Saarland, 66123 Saarbruecken, Germany

**Keywords:** *Streptomyces*, NRPS, heterologous expression, cyclic peptide

## Abstract

The intriguing structural complexity of molecules produced by natural organisms is uncontested. Natural scaffolds serve as an important basis for the development of molecules with broad applications, e.g., therapeutics or agrochemicals. Research in recent decades has demonstrated that by means of classic metabolite extraction from microbes only a small portion of natural products can be accessed. The use of genome mining and heterologous expression approaches represents a promising way to discover new natural compounds. In this paper we report the discovery of a novel cyclic pentapeptide called bonsecamin through the heterologous expression of a cryptic NRPS gene cluster from *Streptomyces albus* ssp. *chlorinus* NRRL B-24108 in *Streptomyces albus* Del14. The new compound was successfully isolated and structurally characterized using NMR. The minimal set of genes required for bonsecamin production was determined through bioinformatic analysis and gene deletion experiments. A biosynthetic route leading to the production of bonsecamin is proposed in this paper.

## 1. Introduction

In the last two decades a considerable number of new small cyclic natural peptides produced by the bacterial genus of *Streptomyces* were discovered and published [1,2,3,4,5,6,7,8]. In nature, those molecules often have either toxic functions or serve the producing organism to coordinate the uptake of metal ions acting as a chelator [9,10]. Several naturally occurring cyclic peptides have proven their potential for use in pharmacotherapy. Remarkable antitumor and antibacterial activity in the recently discovered cyclic peptides chloptosin, hytramycins and mannopeptimycins, was reported by researchers [5,7,11]. Other compounds such as the antitumor agents actinomycin D and romidepsin, or immunosuppressant cyclosporine A, are already established as marketed drugs [12,13,14,15].

Cyclic peptides are often the product of enzymes belonging to the class of nonribosomal peptide synthetases (NRPS). NRPS are large modular enzymes or enzymatic complexes with each module responsible for the incorporation of a single amino acid residue into a nascent peptide chain. The individual modules can be split into separate domains. The typical elongation module contains a minimal set of condensation (C), adenylation (A) and peptidyl carrier (PCP) domains, where the A domain catalyzes the selective activation of an amino acid, the PCP domain holds the activated amino acid or the growing peptide chain and the C domain catalyzes the formation of the amide bond between two PCP-bound amino acid substrates. NRPS-derived molecules can display uncommon structural features, e.g., by incorporation of non-proteinogenic amino acids or when the amino acids are structurally modified by tailoring domains. The chemical diversity of NRPS products is further expanded by the existence of hybrid NRPS clusters [16].

**Figure 1:**
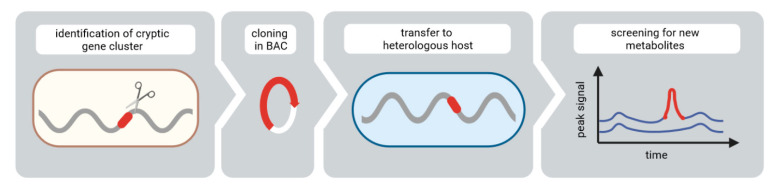
General workflow for compound discovery using heterologous expression.

Recently we reported the successful expression of several cryptic secondary metabolite gene clusters of *Streptomyces albus* ssp. *chlorinus* in the engineered heterologous host strain *Streptomyces albus* Del14. This led to the identification of the biosynthetic genes of the characterized bioactive compounds, albucidin and nybomycin, as well as to the discovery of the new natural products, benzanthric acid, fredericamycin C_2_ and dudomycins [17,18,19,20,21]. In this paper we report the discovery of a new cyclic pentapeptide, bonsecamin, through the heterologous expression of a cryptic NRPS gene cluster of *S. albus* ssp. *chlorinus* in *S. albus* Del14 (Figure 1). Bonsecamin was purified and its structure was elucidated by NMR. The bioinformatic analysis of the putative bonsecamin biosynthetic cluster as well as targeted gene inactivation experiments allowed the determination of the minimal set of genes responsible for the biosynthesis of the compound. A biosynthetic scheme leading to the production of bonsecamin is proposed in this paper.

## 2. Materials and Methods

### 2.1. General Procedures

Table S1 provides an overview of all bacterial strains, BACs and plasmids used in this work. Lysogeny broth (LB) medium was used for cultivation of *Escherichia coli* strains [22]. Soy flour mannitol (MS) agar [23] and tryptic soy broth (TSB; Sigma-Aldrich, St. Louis, MO, USA) were used to grow *Streptomyces* strains. Metabolites were extracted from either liquid DNPM medium (40 g/L dextrin, 7.5 g/L soytone, 5 g/L baking yeast, and 21 g/L MOPS, pH 6.8 as aqueous solution) or defined medium DM (mannitol 5 g/L, amino acid 0.5 g/L, K2PO_4_ 0.5 g/L, MgSO_4_ x 7 H_2_O 0.2 g/L, FeSO_4_ x 7 H_2_O 0.01 g/L). Before inoculation of DM, the cells of the preculture were washed three times with amino-acid-free DM. Amino acids L-lys, L-val, L-ala, D-thr and D-ser were supplied to the defined medium as required by the experimental design. The antibiotics kanamycin, apramycin, ampicillin and nalidixic acid were added when needed.

### 2.2. DNA Isolation and Manipulation

Standard protocols were used to carry out DNA manipulation, transformation into *E. coli* and intergeneric conjugation between *E. coli* and *Streptomyces* [22,23,24]. The BACMAX™ DNA purification kit (Lucigen, Middleton, WI, USA) was used to isolate BAC DNA from a constructed genomic library of *Streptomyces albus* ssp. *chlorinus* NRRL B-24108. Cluster borders were determined by deletion of several genes downstream or upstream from the expected gene cluster with deletion of genes 15 to 28 leading to minimal BAC 2O18_del1 and deletion of genes 1 to 7 leading to BAC 2O18_del2. The genes were replaced by the resistance marker ampicillin through homologous recombination using the RedET system [25]. For amplification of the respective gene cassettes from the plasmid pUC19 PCR reactions were performed. The PCR primer pairs 20200815_1_fw / 20200815_1_rev and 20200815_2_fw / 20200815_2_rev were constructed with overhang regions allowing the site-specific introduction of the cassettes at both sides of the expected bonsecamin gene cluster. The success of the recombination was assessed by restriction mapping and DNA sequencing. The restriction enzymes purchased from ThermoFisher Scientific (Waltham, MA, USA) or New England BioLabs NEB (Ipswich, MA, USA) were used according to the manual. The deletion of three single genes was carried out as described above. To avoid polar effects, additional *Pme*I recognition sites in primers 20201217_1_fw / 20201217_1_rev, 20201217_2_fw / 20201217_2_rev and 20201217_4_fw / 20201217_4_rev allowed the precise removal of the ampicillin resistance gene and religation to BACs 2O18_delKR_delbla, 2O18_delPCP_delbla, and 2O18_delTE2_delbla.

### 2.3. Metabolite Extraction

*Streptomyces* strains were grown in 15 mL of TSB medium in a 100 mL baffled flask for 1 to 2 days. 1 mL of this preculture was used to inoculate 100 mL of production medium in a 500 mL baffled flask. Cultures were incubated for 6 to 7 days at 28°C and 180 rpm in an Infors multitron shaker (Infors AG, Basel, Switzerland) for metabolite production. The metabolites were extracted from the culture supernatant with an equal amount of *n*-butanol, dried at up to 50°C and stored at 4°C.

### 2.4. Mass Spectrometry (MS) Analysis of Metabolites

Prior to MS analysis the extracts were dissolved in methanol. MS experiments were performed on a Dionex Ultimate 3000 UPLC system (ThermoFisher Scientific, Waltham, MA, USA) with PDA detector (stationary phase 100 mm ACQUITY UPLC BEH C18 1.7 µm column (Waters Corporation, Milford, MA, USA), mobile phase: linear gradient of [A] ddH2O + 0.1% formic acid / [B] acetonitrile + 0.1% formic acid, 5% to 95% at a flow rate of 0.6 mL/min). For mass detection the system was further coupled to either an amaZon speed (Bruker, Billerica, MA, USA) or LTQ Orbitrap XL mass spectrometer (ThermoFisher Scientific, Waltham, MA, USA) applying standard settings of positive ionization and a mass range detection of m/z 200 to 2000. The data were analyzed by the softwares Compass Data Analysis 4.1 (Bruker) or Xcalibur 3.0 (ThermoFisher Scientific).

### 2.5. Extract Purification

The crude extracts from the 10 L cultures were dissolved in methanol. Four purification steps were carried out when using DNPM medium for cultivation: (1) Normal Phase (NP) Flash chromatography on an Isolera One system (Biotage, Uppsala, Sweden); stationary phase: SNAP Ultra 50 g (Biotage, Uppsala, Sweden), mobile phase: [A] n-hexane / [B] chloroform / [C] ethyl acetate / [D] methanol in linear gradients of [A]/[B] 10 column volumes (CV), [B]/[C] 15 CV, [C]/[D] 15 CV at a flow of 100 mL/min) was followed by (2) Size Exclusion Chromatography (SEC; stationary phase: Sephadex-LH20; mobile phase: isocratic elution using 100% methanol). Two Reversed Phase (RP) chromatography steps followed on a (3) Waters HPLC system (2545 Binary Gradient module, Waters, Milford, MA, USA); stationary phase: Nucleodur C18 HTec 250/21 5 µm (Macherey-Nagel, Düren, Germany); mobile phase: linear gradient of [A] H_2_O + 0.1% formic acid / [B] methanol + 0.1% formic acid, 5% to 95% [B] for 17 min at flow rate of 20 mL/min, mass detection m/z 430 using software MassLynx (Waters)) and an (4) Agilent Infinity 1200 series HPLC system (stationary phase: Synergi 4 µm Fusion-RP 80 Å 250 × 10 (phenomenex, Torrance, CA, USA); mobile phase: linear gradient of [A] H_2_O + 0.1% formic acid / [B] acetonitrile + 0.1% formic acid, 10% to 50% [B] for 15.5 min at flow rate of 4 mL/min, detection UV 201 nm followed by fraction control on HPLC-MS) yielding 0.7 mg of pure substance. In between all purification steps the fractions containing bonsecamin were pooled, dried (at up to 50°C) and redissolved in methanol.

### 2.6. Structure Elucidation by NMR Spectroscopy, Optical Rotation and Marfey’s Method

NMR spectra were acquired on a Bruker Avance III 700 MHz spectrometer at 298 K equipped with a 5 mm TCI cryoprobe. The chemical shifts (δ) were reported in parts per million (ppm) relative to TMS. As solvents, deuterated DMSO-*d*6 (δH 2.50 ppm, δC 39.51 ppm) from Deutero (Deutero, Kastellaun, Germany) were used. Edited-HSQC, HMBC, ^1^H-^1^H COSY, and N-HSQC spectra were recorded using the standard pulse programs from the TOPSPIN v.3.6 software. Optical rotations were measured using a Perkin Elmer Polarimeter Model 241 (Perkin Elmer, Ueberlingen, Germany).

For Marfey’s method, bonsecamin was hydrolyzed in 100 µL 6 N HCl at 110°C for 1 h. While cooling down, the sample was dried for 15 min under nitrogen, dissolved in 110 mL water and 50 µL each were transferred into 1.5 mL Eppendorf tubes. To the hydrolysate, 20 µL of 1N NaHCO_3_ and 20 µL of 1% L-FDLA or D-FDLA in acetone were added, respectively. The amino acid standards were prepared the same way using L-FDLA only. The reaction mixtures were incubated at 40°C for 90 min at 700 rpm and subsequently quenched with 2N HCl to stop the reaction. The samples were diluted with 300 µL ACN and 1 µL was analyzed by maXis high-resolution LC-QTOF system using aqueous ACN with 0.1 vol% formic acid and an adjusted gradient of 5–10 vol% for 2 min, 10–25 vol% for 13 min, 25–50 vol% for 7 min and 50–95 vol% for 2 min. Detection was carried out at 340 nm.

### 2.7. Genome Mining and Bioinformatic Analysis

The antiSMASH online tool was used to screen the genome of *S. albus* ssp. *chlorinus* (https://antismash.secondarymetabolites.org/#!/start, accessed on 10 July 2021) [26]. Analysis of the genetic data was performed by Geneious software, version 11.0.3 [27]. The genomic sequence of *Streptomyces albus* ssp. *chlorinus* can be accessed in GenBank under VJOK00000000. The Dictionary of Natural Products (DNP) 28.1 was used as reference database of so far characterized metabolites.

## 3. Results and Discussion

### 3.1. Identification and Expression of the NRPS Gene Cluster

**Figure 2:**
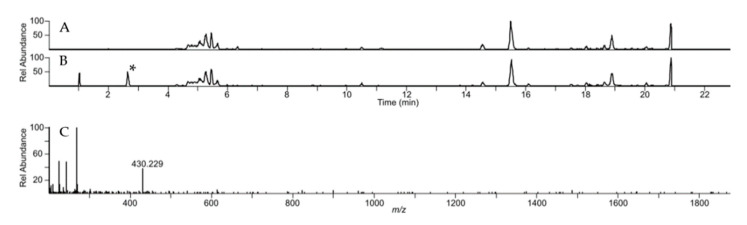
HPLC-MS analysis of bonsecamin production. A- and B-extracted ion chromatograms (430.5 ± 0.5 Da) of crude extracts of *S. albus* 2O18 and *S. albus* Del14, respectively. The new peak observed in the extract of *S. albus* 2O18 is marked with an asterisk (*). C–Mass spectrum of the new peak observed in the extract of *S. albus* 2O18.

Recently, the potential of the strain *Streptomyces albus* ssp. *chlorinus*, as a source of new and undiscovered biomolecules, was demonstrated [17,18,19]. Genome mining of this strain (GenBank accession number VJOK00000000) using antiSMASH software revealed a further uncharacterized biosynthetic gene cluster encoding a putative nonribosomal peptide synthetase (NRPS) [26]. A search in the previously constructed genomic BAC library of *S. albus* ssp. *chlorinus* uncovered a BAC clone 2O18 with the cloned DNA fragment covering the entire NRPS gene cluster. The BAC 2O18 was transferred into the heterologous host *Streptomyces albus* Del14 by conjugation [28]. The obtained exconjugant strain *S. albus* 2O18 as well as the control strain *S. albus* Del14 were cultivated in the production medium DNPM and secondary metabolites were extracted with *n*-butanol. The extracts were analyzed using high resolution LC-MS. The analysis of the peak profile of *S. albus* 2O18 revealed the presence of a new mass peak with an m/z of 430.229 eluting at hydrophilic conditions at the very front of the chromatogram (Figure 2). The identified peak was not detected in the extract of the reference strain without the BAC. The calculated molecular mass of 429.221 [+/−5 ppm] of the identified compound was used for a search in the DNP database of natural products. This survey did not lead to any match with already registered metabolites from bacteria, implying that the structure of the molecule might be new. In order to gain insights into the structure of the identified compound, its purification for NMR analysis was carried out.

### 3.2. Purification and Structure Elucidation

To obtain the identified compound in an amount sufficient for structure elucidation, *S. albus* 2O18 was cultivated in 10L of DNPM medium. The metabolites were extracted with *n*-butanol. The compound was purified from the crude extract using normal-phase, size-exclusion and reversed-phase chromatography. Only a low submilligram amount of pure compound was obtained as a white powder and physically characterized. The optical rotation was determined as ${\left[\alpha \right]}_{D}^{20}\;$−13.5 (c 0.12, MeOH) and the measured λ_max_ (log ε) of the compound was 198 nm (2.11) in 11% ACN/H_2_O + 0.1% formic acid. The dried isolate proved stable for > 1 year when stored at –20 °C and did not show signs of degradation when dissolved in organic solvents (methanol/DMSO). It was further used for structure elucidation by NMR experiments, MS/MS fragmentation and FDLA derivatization (Table 1, Figure S1—S8).

The molecular formula was calculated as C_18_H_31_N_5_O_7_ with 6 degrees of unsaturation corresponding to the monoisotopic mass of 429.222 Da. The analysis of ^1^H NMR in DMSO-*d*6 revealed four doublet NH signals at δH 7.06, 7.84, 7.87 and 8.14, indicating four peptide bonds. The measurement of ^15^N-HSQC confirmed this assumption by showing correlations to δN 114.5, 120.5, 121.7 and 122.4, and revealed an additional NH group at δH 2.31 and δN 48.3 suggesting a secondary amine (Figure S6). Analysis of ^1^H and ^13^C NMR, ^1^H-^1^H COSY and edited-HSQC revealed five amino acids corresponding to valine, two alanines, serine and threonine. The peptide was assigned by long-range HMBC correlations leading to the sequence Ser-Ala-Ala-Val-Thr. The remaining degree of unsaturation indicated a cyclic structure with a ring closure between serine and threonine via the aforementioned amine. This could be concluded from the altered chemical shifts of serine CH-α (δC 62.4, δH 2.80) and threonine CH-β (δC 56.2, δH 3.23). A final proof was provided by key correlations in the ^1^H-^1^H COSY and HMBC spectra. The amine proton at δH 2.31 was determined by a COSY correlation adjacent to CH-α of serine (δH 2.80) and showed strong HMBC correlations to CH-α and CH_3_ of threonine, but not to the carboxyl group. In addition, a COSY correlation of the hydroxyl group of serine (δH 5.01) to CH_2_ β (δH 3.44 and 3.57) was observed, ruling out ring closure by an ester or ether.

In summary, ring closure between serine and CH-α of threonine was established. This will henceforth be referred to as 2, 3-diaminobutanoic acid (DABA). The missing COSY correlation between CH-β of DABA and the amine could be due to a dihedral angle of 90° between δH 2.31 (NH, Ser) and δH 3.23 (CH β, DABA) resulting in a very small 3J coupling constant. The structure was confirmed by MS/MS fragmentation by showing a- and x-ions patterns commonly observed for cyclic structures (Figure S8). The spectral data of bonsecamin are shown in Table 1.

The absolute configuration was determined by Marfey’s method. The peptide was treated with 6N hydrochloric acid at 110°C for 1 h. The hydrolysate and the amino acid standards were derivatized with D- and L-FDLA and analyzed by LC-MS (Figure S7). Alanine and valine were determined in an L-configuration and showed the expected ratio of 2:1. 

**Figure 3:**
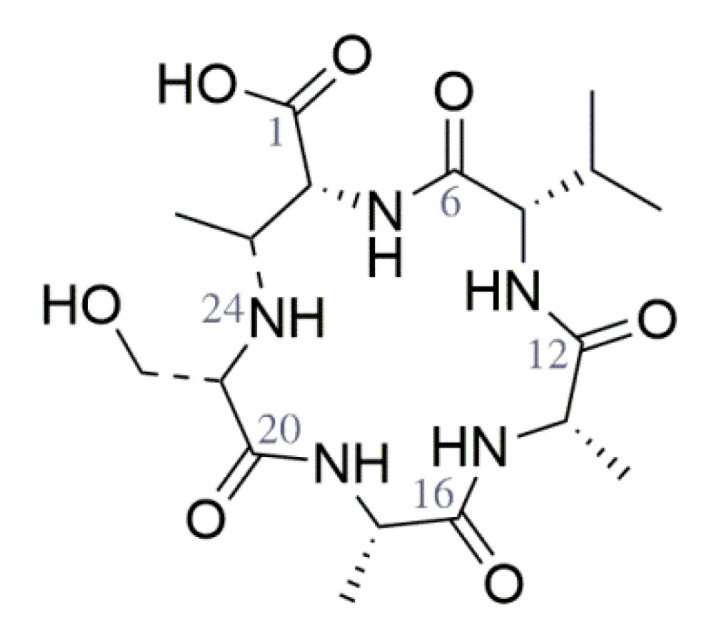
The structure of isolated bonsecamin.

Serine was probably converted to 2,3-hydroxybutanoic acid and did not react with L-FDLA. Thus, its configuration could not be determined. The configuration of DABA was elucidated by the relative method using derivatization with D- and L-FDLA. When derivatized with D-FDLA, DABA showed a shorter retention time compared to L-FDLA, thus it had D-configuration. The absolute structure of bonsecamin is shown in Figure 3. 

### 3.3. Determination of the Minimal Gene Cluster

**Figure 4:**
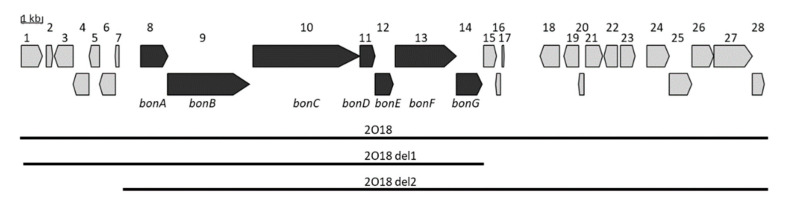
Fragment of the *Streptomyces albus* ssp. *chlorinus* NRRL B-24108 chromosome cloned in BAC 2O18. The genes putatively involved in bonsecamin biosynthesis are highlighted in dark grey. The black bars indicate the chromosomal fragments cloned in BACs 2O18, 2O18_del1 and 2O18_del2 [29].

A sequence analysis of the 35 kb DNA fragment cloned in the BAC 2O18 revealed 28 putative genes (Figure 4). AntiSMASH analysis did not predict any significant homology between the expressed cluster and any other already characterized gene cluster. However, this analysis revealed that a DNA sequence highly similar to genes 8 to 14 is present in a number of *Streptomyces* strains. This implies indirectly that genes 8 to 14 cloned in the BAC 2O18 might correspond to the bonsecamin biosynthetic cluster. Genes 8 to 14 are further assigned as *bonA* to *bonG*. The genes *bonB*, *bon*C and *bonF* encode elements of an NRPS (Table S2). The gene *bonG* shows similarity to an amino acid ligase. The genes *bonD, bonE* and *bonA* encode two putative oxidoreductases and a transporter protein, respectively (Table S2).

To prove the minimal set of genes responsible for bonsecamin synthesis, a set of gene deletion experiments were performed. The genes 15 to 28 which were predicted not to be involved in bonsecamin biosynthesis were deleted in the BAC 2O18 using RedET. The constructed recombinant BAC 2O18_del1 was transferred into the host strain *S. albus* Del14 and the obtained strain *S. albus* 2O18_del1 was tested for bonsecamin production. The results of the HPLC-MS analysis demonstrated that the production of bonsecamin was not affected in the *S. albus* 2O18_del1 strain (Figure S9). This indicates that the genes 15–28 downstream of the *bonG* gene were not involved in bonsecamin production. Since *bonG* encoded a putative amino acid ligase and built an operon with the NRPS gene *bonF*, *bonG* was regarded as the last gene of the biosynthetic cluster (Figure 4). In order to determine the left border of the cluster, genes 1–7 upstream of the *bonA* gene were deleted in BAC 2O18. The constructed BAC 2O18_del2 was transferred into the heterologous host *S. albus* Del14 and the obtained exconjugant strain was analyzed for bonsecamin production. No difference in bonsecamin production between the strains *S. albus* 2O18 and *S. albus* 2O18_del2 was detected (Figure S9). This indicated that the deleted genes 1 to 7 were not involved in the production of bonsecamin. The gene *bonA* encoding a putative transporter was regarded as the first gene involved in bonsecamin production and *bonA* belongs to the same operon as the NRPS-encoding *bonB,* which further supports the assumption that the *bonA* gene constitutes the left border of the cluster. In general, our data indicated that the bonsecamin biosynthetic cluster encompassed the genes *bonA* to *bonG*.

### 3.4. Biosynthesis of Bonsecamin

Bonsecamin is a novel cyclic pentapeptide. The structure of bonsecamin implies that the compound might be synthesized through a linear peptide precursor–Ser-Ala-Ala-Val-Thr. The amino acid residues within the precursor are linked via conventional amide bonds. To generate the mature bonsecamin the linear precursor peptide likely undergoes intramolecular dehydrative cyclization. During this modification step the side chain of the threonine was joined with the free amino group of the serine residue.

**Figure 5:**
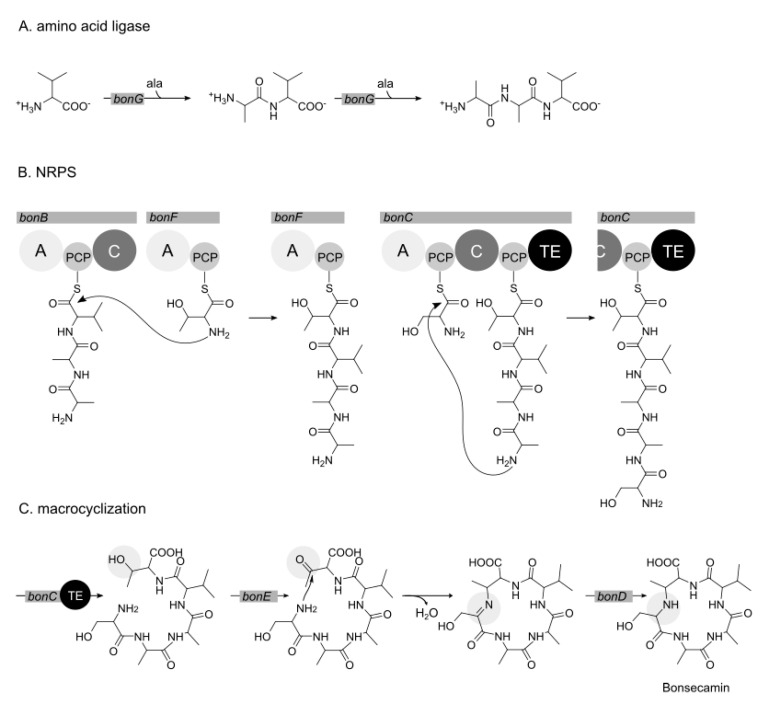
Proposed biosynthetic scheme for bonsecamin production. A. Formation of the Ala-Ala-Val tripeptide precursor catalyzed by the putative alanine ligase encoded by *bonG*. B. Conversion of the tripeptide intermediate into the linear pentapeptide precursor catalyzed by the NRPS encoded by *bonB*, *bonF* and *bonC*. C. Cyclization of the linear bonsecamin precursor catalyzed by the products of *bonE* and *bonD*.

The predicted minimal gene cluster for bonsecamin production consists of seven open reading frames from *bonA* to *bonG*. The *bonA* gene encodes a putative transporter. The genes *bonD* and *bonE* encode putative dehydrogenases and are presumably involved in the tailoring steps of the bonsecamin biosynthesis. The remaining four genes *bonB*, *bonC*, *bonF* and *bonG*, which encode putative elements of NRPS and an alanine ligase, might be involved in the biosynthesis of the linear bonsecamin precursor. The analysis of the NRPS genes revealed that *bonF* encodes A and PCP domains; *bonB* encodes A, PCP and C domains; and *bonC* encodes A, PCP, C and TE domains (Figure 5). This domain organization indicates that only three of five amino acid residues of bonsecamin were incorporated by the encoded NRPS. The A domains encoded by *bonC*, *bonB* and *bonF* were predicted to have substrate specificity towards the amino acids serine, valine and threonine, respectively. This prediction is in accordance with the amino acid composition of bonsecamin: Ser-Ala-Ala-Val-Thr. Two alanine residues are therefore expected to be incorporated into the bonsecamin precursor in an NRPS-independent manner. The putative alanine ligase encoded by the *bonG* gene might be responsible for the alanine incorporation.

Taking into consideration the structure of the compound, domain organization and the predicted substrate specificity of the NRPS, the following bonsecamin biosynthetic scheme is proposed. The first step in the biosynthesis is the assembly of the Ala-Ala-Val tripeptide catalyzed by a putative alanine ligase encoded by *bonG*. The enzyme uses valine as a starter substrate and carries out two rounds of alanine ligation. The participation of amino acid ligases in the biosynthesis of natural products was reported before [30]. Amino acid ligases usually attach small, non-polar amino acids to the amino group of the substrate by forming an amide bond. The enzymes are characterized as being quite specific for their extension unit (here alanine), but exhibit only little substrate specificity for the starter substrate, which is extended [31]. The relaxed substrate specificity might explain the attachment of the alanine residue to the valine in the first elongation step and then to the Ala-Val dipeptide in the second elongation step.

The conversion of the synthesized Ala-Ala-Val tripeptide into the linear bonsecamin precursor is proposed to be catalyzed by the NRPS genes encoded within the cluster. The attachment of the threonine residue to the tripeptide is supposedly catalyzed by the products of *bonB* and *bonF*, which together encode the starter module and the first elongation module of the bonsecamin NRPS. The tripeptide is probably activated by the A domain of *bonB,* presumably specific for valine, and loaded on the corresponding PCP domain (Figure 5). The activated tripeptide is then elongated with threonine which is activated by the A domain of *bonF* and bound to its PCP domain, leading to the Ala-Ala-Val-Thr tetrapeptide (Figure 5).

To finalize the assembly of the linear pentapeptide precursor, an amide bond between the amino group of the serine and the carboxyl group of the alanine within the tetrapeptide product needs to be formed. We propose that the product of *bonC* catalyzes this reaction. *BonC* contains a canonical starter module with A and PCP domains and a shortened termination module with C, PCP and TE domains. The termination module of *bonC* lacks an A domain. The A domain of the starter module is predicted to activate serine and to load it on the corresponding PCP domain. To elongate the serine residue with the tetrapeptide the latter needs to be loaded on the PCP domain of the termination module of *bonC*. Since the termination module of *bonC* does not contain any A domain, we propose that the tetrapeptide is transferred from the PCP domain of *bonF* on the PCP domain of the termination module of *bonC*. The mechanism of this assumed transesterification step remains elusive. The condensation of the serine residue with the tetrapeptide to yield the linear pentapeptide Ser-Ala-Ala-Val-Thr is catalyzed by the C domain of *bonC*. The linear pentapeptide precursor is proposed to be released through the action of a dedicated TE domain within the termination module of *bonC* (Figure 5).

The released linear pentapeptide precursor needs to be cyclized to yield mature bonsecamin. The cylization of bonsecamin occurs not through conventional amide bonds but through the secondary amine, which is formed between the side chain of the threonine and the amino group of the serine. We suppose that the conversion of the linear precursor into bonsecamin is catalyzed by the standalone enzymes encoded by *bonD* and *bonE*. Both enzymes were annotated to possess redox function. We assume that the first step towards cyclization is the oxidation of the hydroxyl group at the β carbon of the threonine, leading to a keto group with reactivity superior to the carboxylic acid carbon. This step might be catalyzed by the product of *bonE* which shows sequence similarity to 3-hydroxybutyrate dehydrogenase and 3-ketoacyl-ACP reductase–enzymes which catalyze similar reactions. Then the amino group of the serine residue reacts with the generated keto group under release of a water molecule to give an imine intermediate. Peptide macrocyclizations leading to imines are unusual, but have been described before [32,33,34]. The process of cyclization seems to happen spontaneously [32]. The generated imine group within the cyclized precursor is further reduced to a secondary amine leading to mature bonsecamin. We suppose that the final reduction step is catalyzed by the product of *bonD* which shows homology to the product of *lgrE*, involved in the reduction of linear gramicidin precursors [35].

The proposed scheme for bonsecamin biosynthesis includes a number of unusual enzymatic steps. In order to validate the proposed biosynthetic pathway, the inactivation of the *bonE* gene as well as the deletion of the second PCP domain or TE domain within *bonC* were undertaken and led to complete cessation of bonsecamin production (Figure S9). No putative derivatives or precursors of the compound could be detected in the culture broth of the engineered strains.

## 4. Conclusions

In this article we reported the identification of the new compound bonsecamin after successful heterologous expression of a cryptic NRPS cluster of *S. albus* ssp. *chlorinus* NRRL B-24108. Bonsecamin is a cyclic pentapeptide with a secondary amine moiety formed between the side chain of a threonine and the amino group of a serine. The identified minimal set of biosynthetic genes indicated that bonsecamin was a result of the interplay between an amino acid ligase and a nonlinear NRPS.

## Figures and Tables

**Table 1:** NMR data of bonsecamin in DMSO-*d*6.

Pos.		δC/δN Type	δH, mult. (J in Hz)	COSY	Key HMBC (H-)
2,3-DABA	1	170.5*, C			
	2	58.53, CH	3.96, dd (7.5, 2.5)	3, 5	1
	3	56.23, CH	3.23, m	2, 4	23
	4	13.41, CH3	0.52, d (6.3)	3	2
	5	122.4, NH	7.06, d (7.5)	2	1, 6
Val	6	169.6*, C			
	7	58.40, CH	4.01, t (9.1)	8, 11	6, 12
	8	30.24, CH	1.96, m	7, 9, 10	
	9	18.39, CH3	0.83, d (7.0)	8	7
	10	19.44, CH3	0.84, d (7.0)	8	7
	11	114.5, NH	7.84, d (9.1)	7	12
Ala	12	172.2*, C			
	13	50.67, CH	4.21, dq (8.6, 7.5)	14, 15	12, 16
	14	17.46, CH3	1.32, d (7.5)	13	12
	15	120.5, NH	8.14, d (8.6)	13	16
Ala	16	169.8*, C			
	17	50.56, CH	4.08, quin (7.2)	18, 19	16, 20
	18	17.23, CH3	1.24, d (7.2)	17	16
	19	121.7, NH	7.87, d (7.2)	17	16
Ser	20	172.9*, C			
	21	62.38, CH	2.80, m	22, 24	3, 20
	22	61.04, CH2	3.44, m	21, 23	
			3.57, m		
	23	----, OH	5.01, m	22	
	24	48.3, NH	2.31, d (5.5)	21	2, 4, 20, 21, 22

*Not visible in the 13C NMR spectrum. The value was taken from the HMBC.

## Data Availability

The sequenced genome of *S. albus* ssp. *chlorinus* is available at GenBank under accession number VJOK00000000.

## Supplementary Materials

The following are available online at https://www.mdpi.com/article/10.3390/microorganisms9081640/s1: Table S1: Strains, BACs, plasmids and primers used in this work; Table S2: Putative products of the genes in the DNA fragment encoding bonsecamin production; Figure S1: ^1^H NMR spectrum (700 MHz, DMSO-d6) of bonsecamin. Figure S2: ^13^C NMR spectrum (700 MHz, DMSO-d6) of bonsecamin. Figure S3: ^1^H-^1^H COSY spectrum (700 MHz, DMSO-d6) of bonsecamin. Figure S4: Edited-HSQC spectrum (700 MHz, DMSO-d6) of bonsecamin. Figure S5: HMBC spectrum (700 MHz, DMSO-d6) of bonsecamin. Figure S6: ^15^N-HSQC spectrum (700 MHz, DMSO-d6) of bonsecamin. Figure S7: LC-MS chromatograms of hydrolyzed bonsecamin derivatized with D- or L-FDLA and the amino acid (aa) references derivatized with L-FDLA. Figure S8: MS/MS fragmentation of bonsecamin. Figure S9: Production of bonsecamin in S. albus Del14 mutant after gene deletion experiments.
