# Supplementary material for: Bonsecamin: A New Cyclic Pentapeptide Discovered through Heterologous Expression of a Cryptic Gene Cluster
Source: Microorganisms. 2021 Jul 31;9(8):1640. doi: 10.3390/microorganisms9081640 (PMC8400726; doi:10.3390/microorganisms9081640)
Supplement: Supplementary file 1 [file microorganisms-09-01640-s001.zip › microorganisms-1303944-supplementary.pdf]

microorganisms

MDPI

Supplementary

Bonsecamin: A New Cyclic Pentapeptide Discovered through Heterologous Expression of a Cryptic Gene Cluster

Constanze Lasch [1], Marc Stierhof [1], Marta Rodríguez Estévez [1], Maksym Myronovskyi [1], Josef Zapp [2] and Andriy Luzhetskyy [1,3,*]

[1] Pharmaceutical Biotechnology, Saarland University, Saarbruecken, Germany; constanze.lasch@uni-saarland.de (C.L.), m.stierhof@t-online.de (M.S.), marta.rodriguezestevez@uni-saarland.de (M.R.), maksym.myronovskyi@uni-saarland.de (M.M.), a.luzhetskyy@mx.uni-saarland.de (A.L.)
[2] Pharmaceutical Biology, Saarland University, Saarbruecken, Germany; j.zapp@mx.uni-saarland.de (J.Z.)
[3] Helmholtz Institute for Pharmaceutical Research Saarland, Saarbruecken, Germany
* Correspondence: a.luzhetskyy@mx.uni-saarland.de; +49 681 302 70200 (A.L.)

Supplementary.

Table S1. Strains, BACs, plasmids and primers used in this work.

Material	Purpose
**A. Bacterial strains**	
Streptomyces albus Del14	heterologous host [1]
Escherichia coli GB05 RedCC	cloning host [Helmholtz-Institut für Pharmazeutische Forschung Saarland (HIPS)]
Escherichia coli ET12567 pUB307	alternate host intergeneric conjugation [2]
**B. BACs**	
2O18	heterologous expression of NRPS cluster
2O18_del1	determination downstream border of NRPS cluster
2O18_del2	determination upstream border of NRPS cluster
2O18_delKR_delbla	single gene inactivation
2O18_delPCP_delbla	single gene inactivation
2O18_delTE2_delbla	single gene inactivation
**C. Plasmids**	
pUC19	ampicillin resistance marker
**D. PCR primer Red/ET**	
20200815_1_fw [2O18_del1]	TAGTCCAGCGTCATCAGCGGGGCGTCCGAGGCACTGCGGACCACGAGGCGCGTCAGGTGGCACTTTTCG
20200815_1_rev [2O18_del1]	TCCGACGGCGGGCGGCCCCGCACTAGGCTCGCCGCCATGACGGACGTCGACTTTTCTACGGGGTCTGAC
20210315_1_fw [2O18_del2]	CTATCGTCGCCACGCCTTGGTGCACGGGAAATCCGGTGTGATGCCGGTGCCGTCAGGTGGCACTTTTCG
20200815_2_rev [2O18_del2]	CACTGGATGCCCAGGCAGGGGGTACGCAGCATGACCGAGGAGGACGCGGCCTTTTCTACGGGGTCTGAC
20201217_1_fw [2O18_delKR_delbla]	GCTGGTGAACCCGCCGTCGACGGTGACCGTGGAGCCGGTCACCTGGCGGGAGTTTAAACCGTCAGGTGGCACTTTTCG

20201217_1_rev [2O18_delKR_delbla]	GGAGTGCTCACCGCGGCGCCGCTCGCGGGCAAGGCCGCCGTCATCACGGGTTTAAACGACTTTT CTACGGGGTCTGAC
20201217_2_fw [2O18_delPCP_delbla]	GGCCAGGGCGGCCAGTTCGCCCAGCCGCGGGATGCGGGTGAGGTCGGTGAAGTTTAAACCGTC AGGTGGCACTTTTCG
20201217_2_rev [2O18_delPCP_delbla]	CGCGCGGTCTGGCAGCAGATCCTGGGGCTGACGGCGGAGGAGATCGGTGGTTTAAACGACTTT TCTACGGGGTCTGAC
20201217_4_fw [2O18_delTE2_delbla]	CAGTTCCGCGGTCGCGGCCGGATTGCCGCGCACGAAGTAGTGGCCGCCCGAGTTTAAACCGTC AGGTGGCACTTTTCG
20201217_4_rev [2O18_delTE2_delbla]	TGCGTGCCGTATCCGTGCGGGCACCCGGTCAACTTCAAACCGCTGGCCGGTTTAAACGACTTTT CTACGGGGTCTGAC

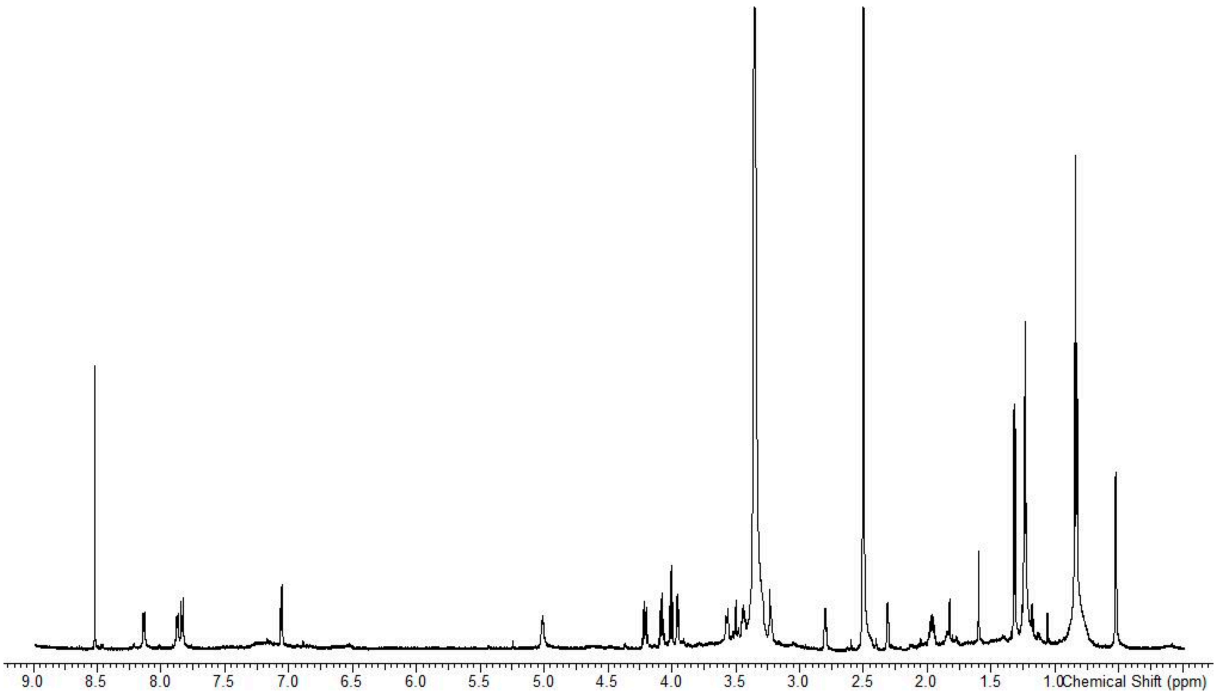

Figure S1: ^{1}H NMR spectrum (700 MHz, DMSO-*d6*) of bonsecamin.

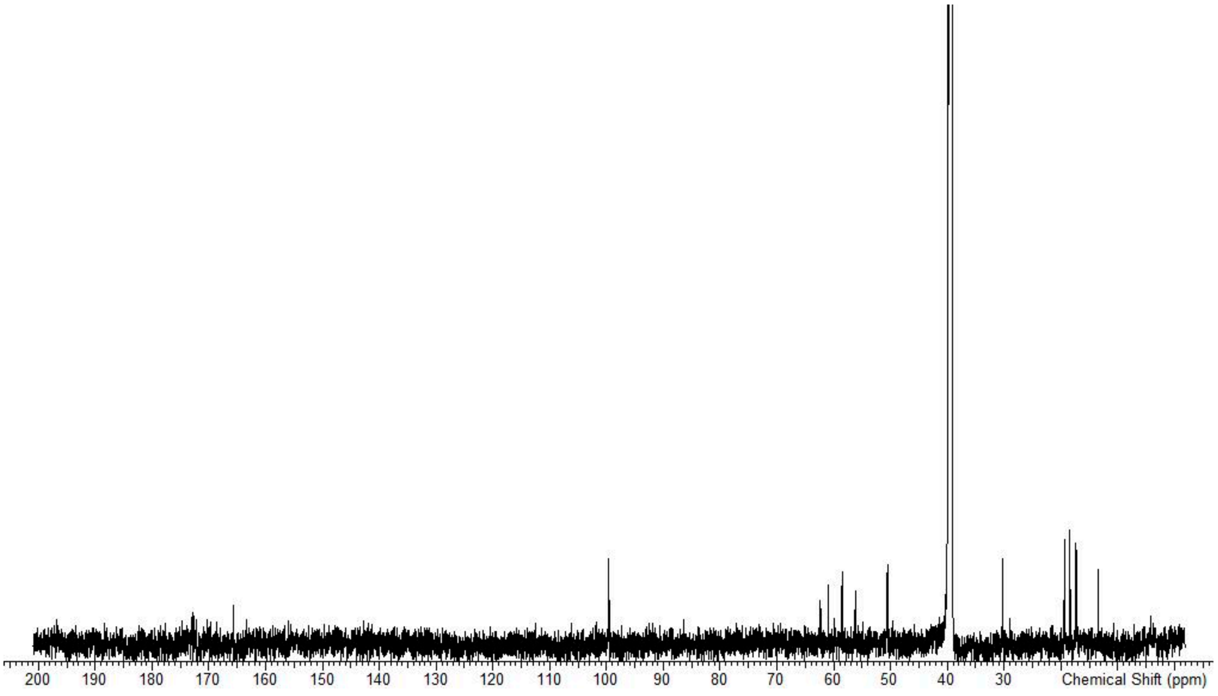

Figure S2: ^{13}C NMR spectrum (700 MHz, DMSO-*d6*) of bonsecamin.

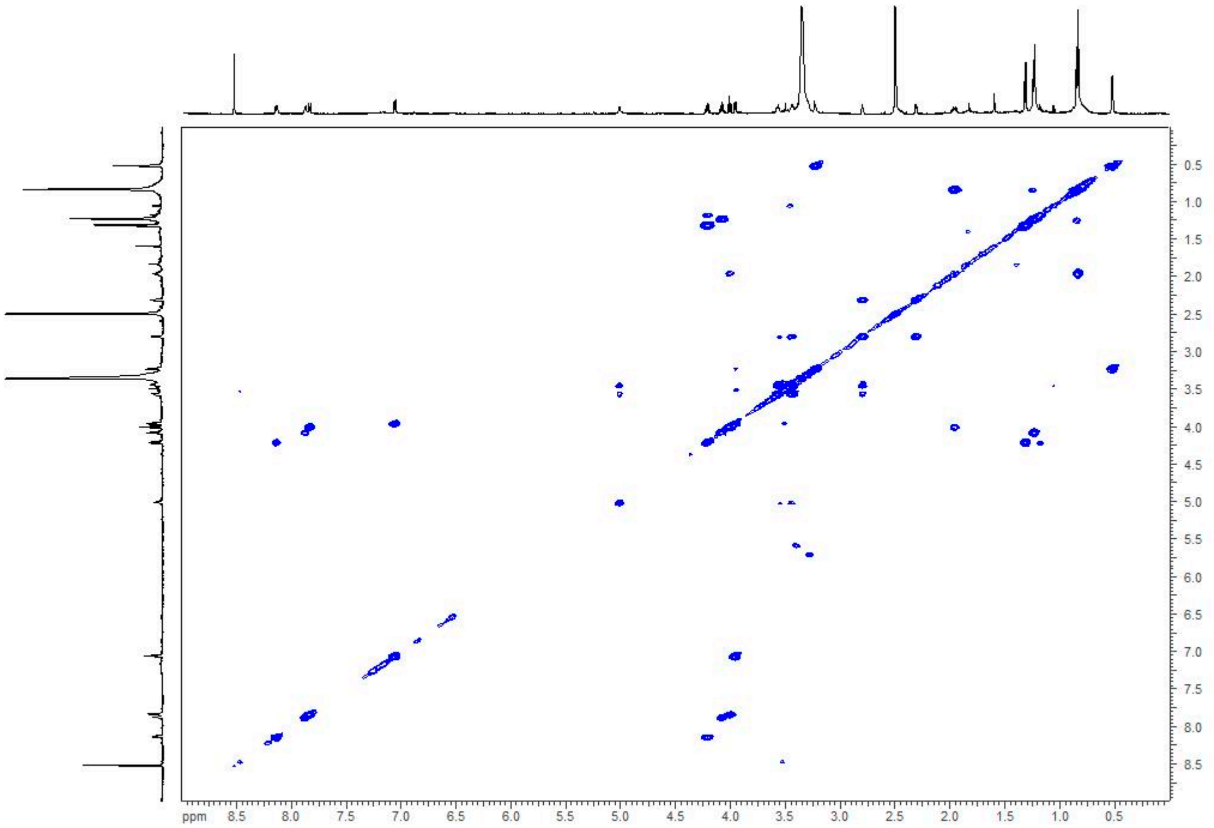

Figure S3: ^{1}H-^{1}H COSY spectrum (700 MHz, DMSO-*d6*) of bonsecamin.

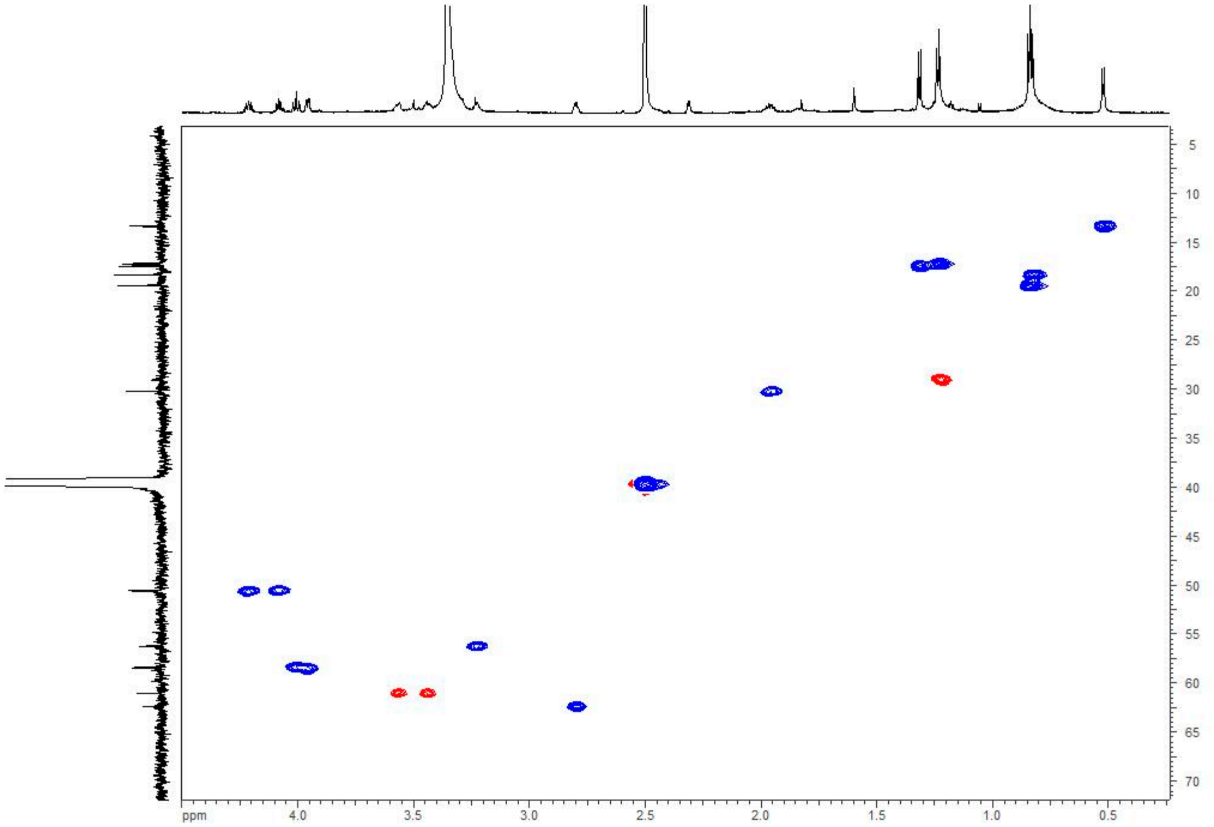

Figure S4: Edited-HSQC spectrum (700 MHz, DMSO-*d6*) of bonsecamin.

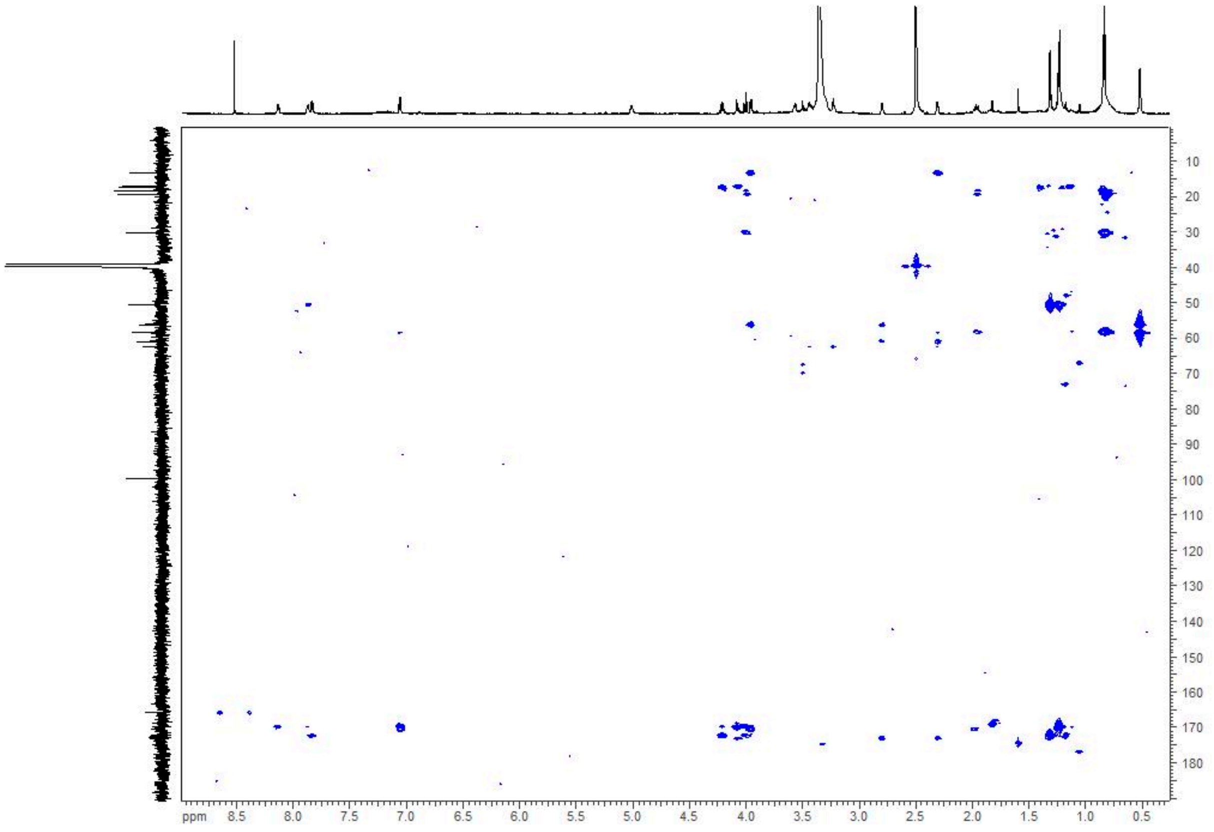

Figure S5: HMBC spectrum (700 MHz, DMSO-*d*6) of bonsecamin.

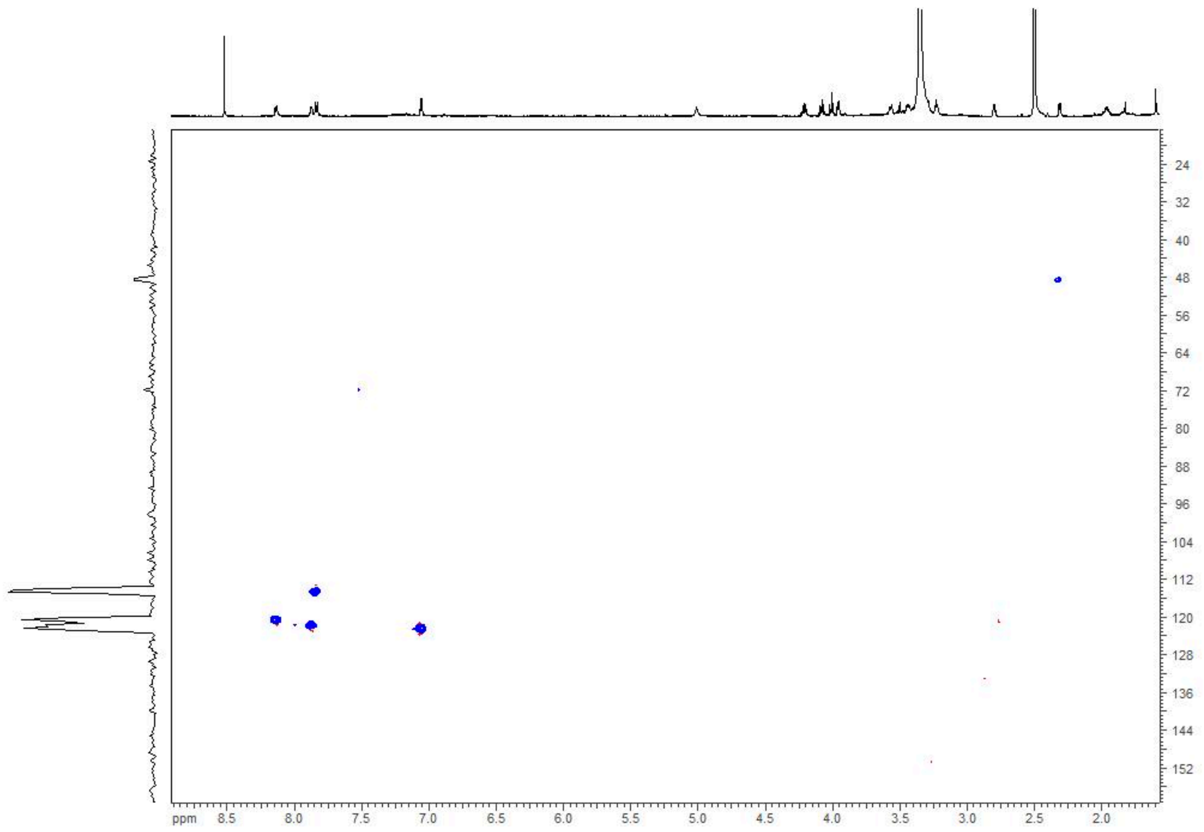

Figure S6: ^{15}N-HSQC spectrum (700 MHz, DMSO-*d*6) of bonsecamin.

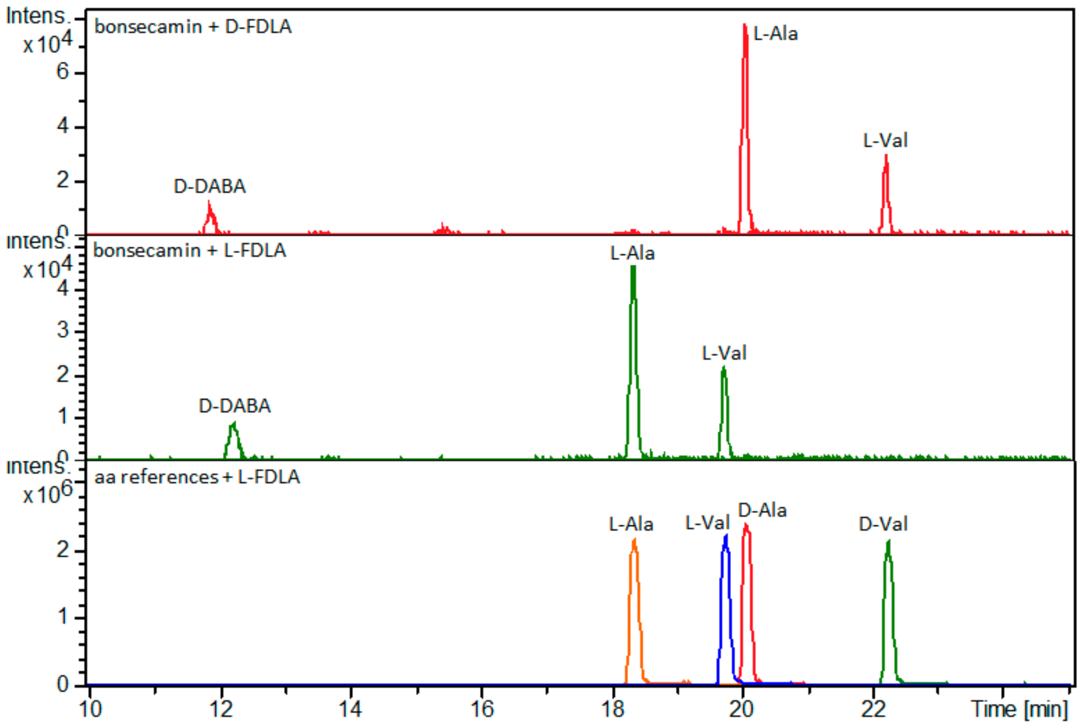

Figure S7: LC-MS chromatograms of hydrolyzed bonsecamin derivatized with D- or L-FDLA and the amino acid (aa) references derivatized with L-FDLA.

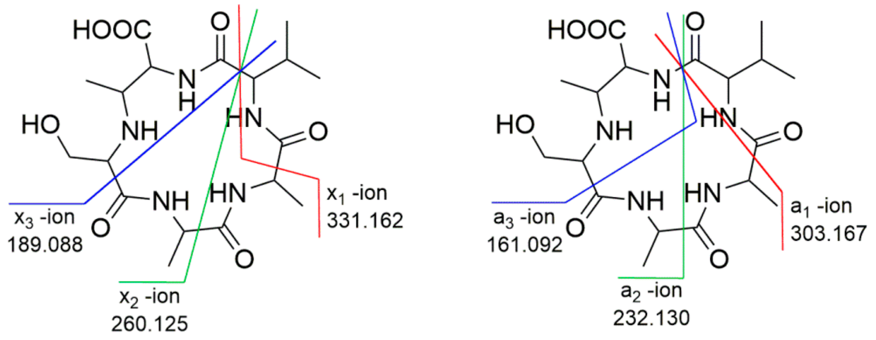

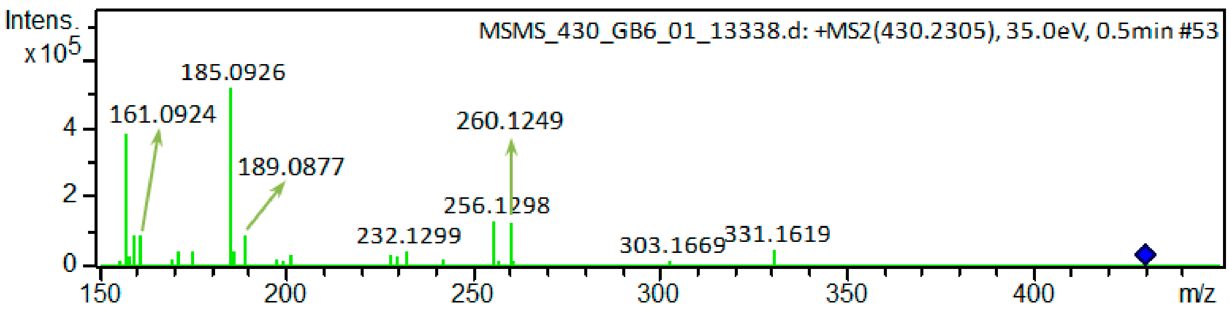

Figure S8: MS/MS fragmentation of bonsecamin.

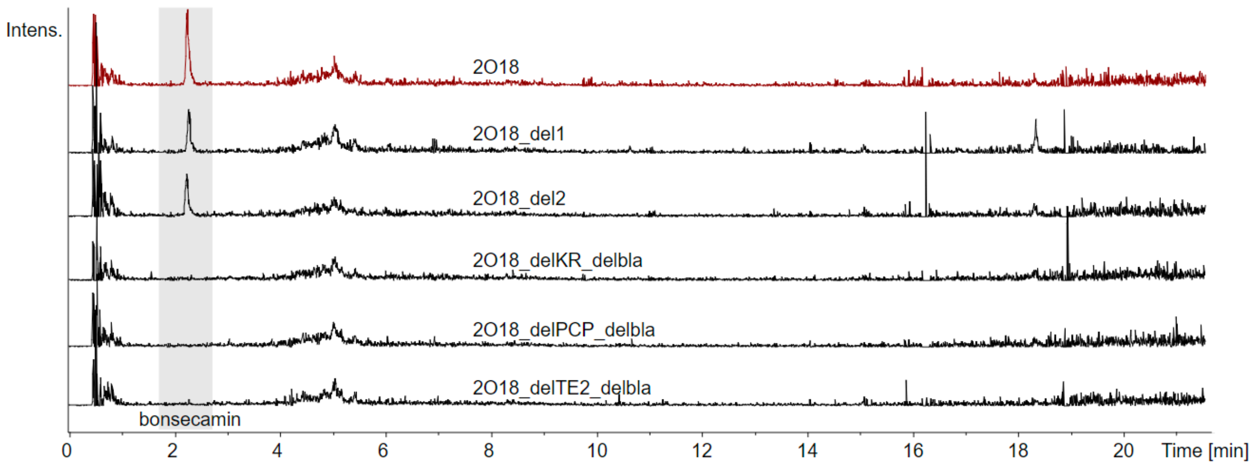

Figure S9. Production of bonsecamin in *S. albus* Del14 mutant after gene deletion experiments. EIC extracted for masses [430-431].

Table S2. Putative products of the genes in the DNA fragment encoding bonsecamin production.

gene #	locus tag	putative product
1	SACHL_05130	Catalase
2	SACHL_05120	hypothetical protein
3	SACHL_05110	Cobalt import ATP-binding protein CbiO
4	SACHL_05100	Cobalt transport protein CbiQ
5	SACHL_05090	Cobalt transport protein CbiN
6	SACHL_05080	Cobalt transport protein CbiM
7	SACHL_05070	-
8 [*bonA*]	SACHL_05060	enterobactin exporter EntS
9 [*bonB*]	SACHL_05050	Tyrocidine synthase 3 - val
10 [*bonC]*	SACHL_05040	Tyrocidine synthase 3 - ser
11 [*bonD*]	SACHL_05030	Linear gramicidin dehydrogenase LgrE
12 [*bonE*]	SACHL_05020	(-)-trans-carveol dehydrogenase
13 [*bonF*]	SACHL_05010	Dimodular nonribosomal peptide synthase - thr
14 [*bonG*]	SACHL_05000	Alanine-anticapsin ligase BacD
15	SACHL_04990	hypothetical protein
16	SACHL_04980	hypothetical protein
17	SACHL_04970	-
18	SACHL_04960	P-aminobenzoate N-oxygenase AurF
19	SACHL_04950	hypothetical protein
20	SACHL_04940	hypothetical protein
21	SACHL_04930	hypothetical protein
22	SACHL_04920	CGNR zinc finger
23	SACHL_04910	(S)-2-haloacid dehalogenase
24	SACHL_04900	Putative phenylalanine aminotransferase
25	SACHL_04890	All-trans-nonaprenyl-diphosphate synthase (geranyl-diphosphate specific)
26	SACHL_04880	prenyltransferase
27	SACHL_04870	Squalene-hopene cyclase
28	SACHL_04860	2-octaprenyl-3-methyl-6-methoxy-1,4-benzoquinol hydroxylase

[1] M. Myronovskyi, B. Rosenkränzer, S. Nadmid, P. Pujic, P. Normand, A. Luzhetskyy, Generation of a cluster-free Streptomyces albus chassis strains for improved heterologous expression of secondary metabolite clusters, Metab. Eng. 49 (2018) 316–324. https://doi.org/10.1016/j.ymben.2018.09.004.

[2] F. Flett, V. Mersinias, C.P. Smith, High efficiency intergeneric conjugal transfer of plasmid DNA from Escherichia coli to methyl DNA-restricting streptomycetes, FEMS Microbiol. Lett. 155 (2006) 223–229. https://doi.org/10.1111/j.1574-6968.1997.tb13882.x.
